# Supplementary material for: The interactome of intact mitochondria by cross-linking mass spectrometry provides evidence for coexisting respiratory supercomplexes
Source: Mol Cell Proteomics. 2017 Dec 8;17(2):216–32. doi: 10.1074/mcp.RA117.000470 (PMC5795388; doi:10.1074/mcp.RA117.000470)
Supplement: Supplemental Data [file supp_RA117.000470_133922_0_supp_23978_fzffwf.zip › spectra_annotation/mito_DR_spectra_annotation/5-1-1-1-4-1.pdf]

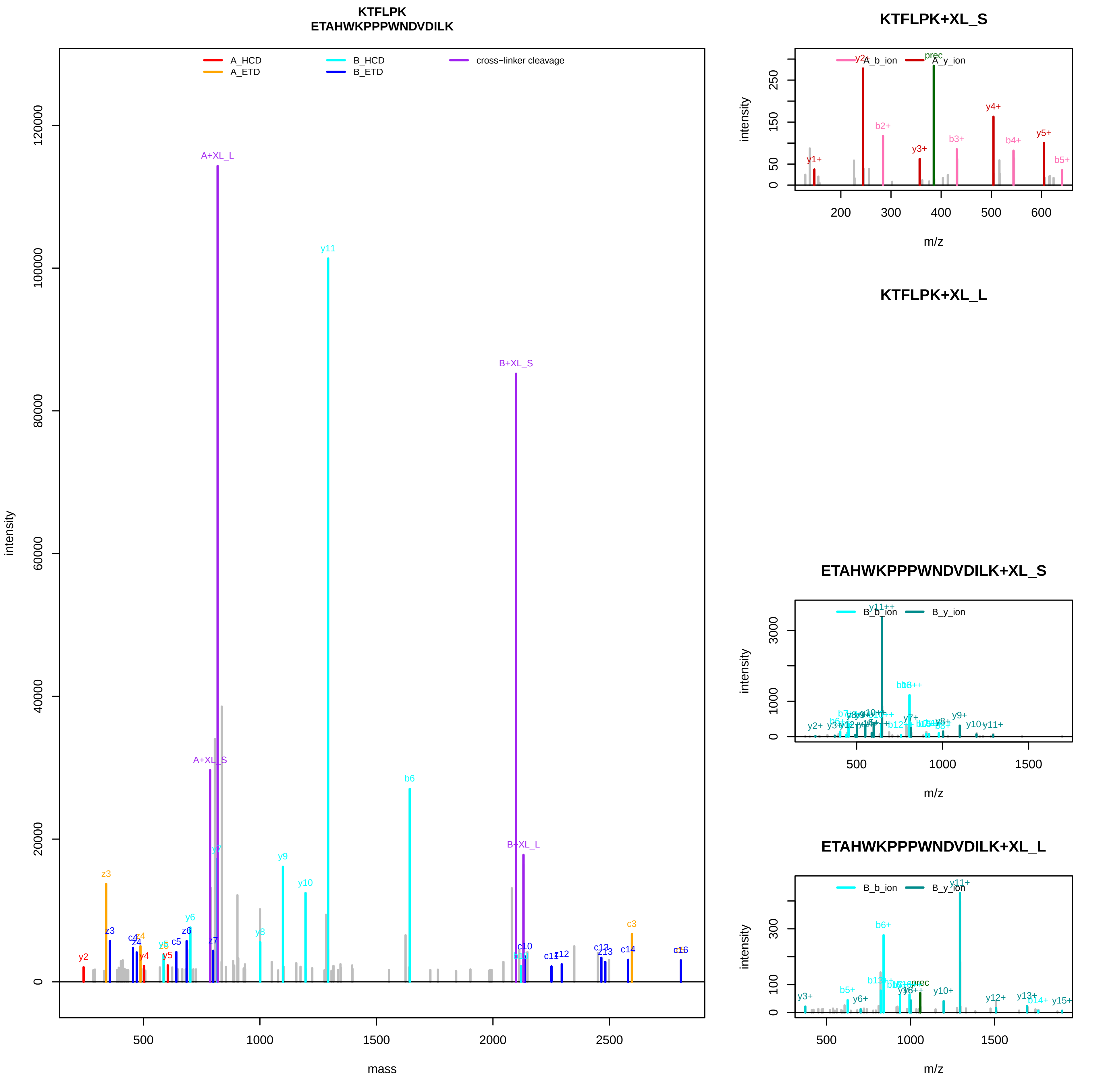
KTFLPK
ETAHWKPPPWNDVDILK
A_HCD
A_ETD
B_HCD
B_ETD
cross−linker cleavage
A+XL_L
y11
B+XL_S
A+XL_S
b6
B+XL_L
y9
y10
y7
z3
c3
c16
intensity
mass
KTFLPK+XL_S
A_b_ion
A_y_ion
prec
y2+
b2+
y4+
y3+
b3+
b4+
y5+
y1+
b5+
m/z
KTFLPK+XL_L
ETAHWKPPPWNDVDILK+XL_S
B_b_ion
B_y_ion
y11++
y9+
y10+
y11+
m/z
ETAHWKPPPWNDVDILK+XL_L
B_b_ion
B_y_ion
y11+
b6+
b5+
y6+
y3+
y10+
y12+
y13+
b14+
y15+
m/z
